# Repeat Prolonged Chlorination at Low Dose Induces Chlorine Tolerance in *Legionella pneumophila* via Viable but Non-culturable State

**DOI:** 10.1021/envhealth.5c00360

**Published:** 2025-10-06

**Authors:** Xiaofei Yuan, Yan Zheng

**Affiliations:** † State Key Laboratory of Soil Pollution Control and Safety, School of Environmental Science and Engineering, Southern University of Science and Technology, Shenzhen 518055, China; ‡ Research Centre for Life Science Computing, 559075 Zhejiang Laboratory, Hangzhou 311100, China; § Guangdong Provincial Key Laboratory of Soil and Groundwater Pollution Control, School of Environmental Science and Engineering, Southern University of Science and Technology, Shenzhen 518055, China

**Keywords:** chlorine tolerance, viable but non-culturable (VBNC) state, chlorine-resistant bacteria, resuscitation, *Legionella*, chloramine-T

## Abstract

Chlorine-resistant *Legionella pneumophila*, the causative agent of fatal pneumonia, poses a major global public health threat. However, the mechanistic basis for this pathogen’s resistance evolution remains elusive. Our study demonstrates that a minimally dormant or the early stages of viable but non-culturable (VBNC) state of bacteria is critical to chlorine tolerance development, representing an initial stage of resistance acquisition. Prolonged low-dose chlorination (12 h exposure to 2 mg/L chloramine-T) induced VBNC transformation in *L. pneumophila*, with a resuscitation lag time ∼10-fold longer than that of parallel nonchlorinated but starved bacteria. Upon resuscitation, secondary chlorination (3 or 12 h) of stationary-phase cells maintained growth rates similar to those of untreated cells, but with significantly shortened lag times (to ∼26 and ∼41 h, vs ∼40 and ∼47 h for single-chlorinated groups, with *p* < 0.01 for the 3 h treatment). Flow cytometry showed that rechlorinated groups had a higher proportion of active cells and a lower proportion of death-analogous cells than single-chlorinated counterparts, indicating the emergence of a chlorine-tolerant subpopulation. These findings directly link the VBNC state to bacterial resistance evolution, underscoring the urgent need to investigate dormant bacteria to control antimicrobial resistance spread in water systems.

## Graphical abstract

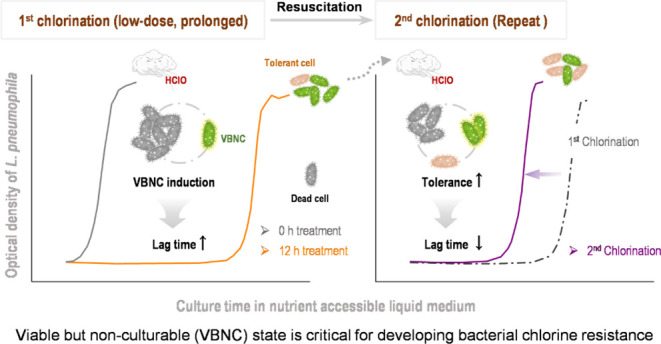

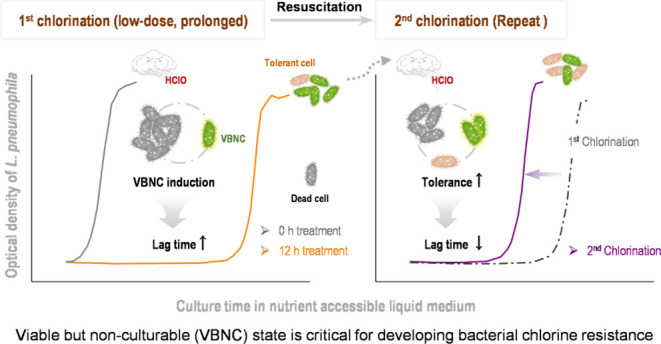

## 1 Introduction

The viable but non-culturable (VBNC) state refers to a dormant-like state in response to environmental stress, in which bacteria maintain low metabolic activity but cannot form colonies on routine nutrient agar plate culture under standard laboratory conditions.^1,2^ This physiological adaptation is recognized as a survival strategy employed by bacteria to counteract stressors^3,4^ such as nutrient deprivation,^5^ extreme temperatures,^6^ sublethal chlorine exposure,^7^ and other growth-restrictive conditions. VBNC bacteria regulate associated gene expression strategies to survive these harsh environmental challenges.^8,9^ However, due to their laboratory uncultivability, VBNC bacteria are often misidentified as nonviable cells,^3,10^ resulting in the under-characterization that may pose severe health consequences. This is because pathogenic species can still synthesize virulence proteins or retain antibiotic-resistant genes in the VBNC state.^1,11,12^ When the VBNC cells are resuscitated under certain conditions, the revived virulence and antibiotic resistance threaten public health.^13,14^ Specifically, VBNC cells could serve as a reservoir of antimicrobial resistance genes to facilitate horizontal gene transfer, promoting the emergence of antibiotic-resistant superbugs,^15^ a process that may be accelerated through the “lagging response” phenomenon.^16^

Chlorination is one of the most successful public health interventions in human history. However, it is associated with unintended consequences, including the formation of chlorine disinfection byproducts^17,18^ and the selection of chlorine-resistant bacteria (CRB).^19^ Of particular concern in this context is the opportunistic waterborne pathogen *Legionella pneumophila* (*L. pneumophila*), which is widespread in natural and artificial aquatic environments^20^ and causes atypical pulmonary infections with a high mortality rate (8–12%) among vulnerable populations (aged ≥ 50 years).^21^ Despite its chlorine sensitivity,^22^ the incidence rate of Legionnaires’ disease is rising in both the USA^23^ and Europe.^24^ *L. pneumophila* has a high tendency to develop into CRB in engineered water systems.^19^ Sublethal chlorine exposure (2 mg/L sodium hypochlorite) has been shown to trigger protective cellular adaptations in *L. pneumophila*, enhancing chlorine resistance.^25^ Low-level chlorination of drinking water was found to not only induce VBNC *Escherichia coli* (0–10^2^ cells/100 mL),^26^ but also to increase antibiotic tolerance.^8,9^

Building on these observations of chlorine-induced adaptations or tolerance, we hypothesize that the VBNC state is critical for chlorine resistance development, aiming to bridge the mechanistic knowledge gap regarding *Legionella*’s acquired chlorine tolerance.^22^ To test this hypothesis, we designed repeated low-dose chlorination experiments that are often encountered in real-world applications of chlorination: sequential chlorination (aimed at generating resuscitable VBNC *L. pneumophila*), resuscitation, and rechlorination of *L. pneumophila* (Figure 1). Chloramine-T trihydrate (CAT), a mild yet highly stable oxidative disinfectant with proven efficacy as an alternative to sodium hypochlorite,^27,28^ was chosen as the chlorinating agent for its stability required to compare results across a wide range of chlorination duration (0–64 h), and for the reason that *L. pneumophila* exhibits exceptional sensitivity to CAT.^29^ VBNC resuscitation was performed in nutrient Buffered Yeast Extract (BYE) liquid medium at 37 °C. Key parameters, including growth rate (μ), lag time (τ), and flow cytometry (FCM)-identified subpopulations, were analyzed in comparison with untreated bacteria to assess the development of chlorine tolerance in *L. pneumophila* following VBNC induction. Finally, the implications of these findings for water utility practices and CRB dissemination are discussed.

**Figure 1:**
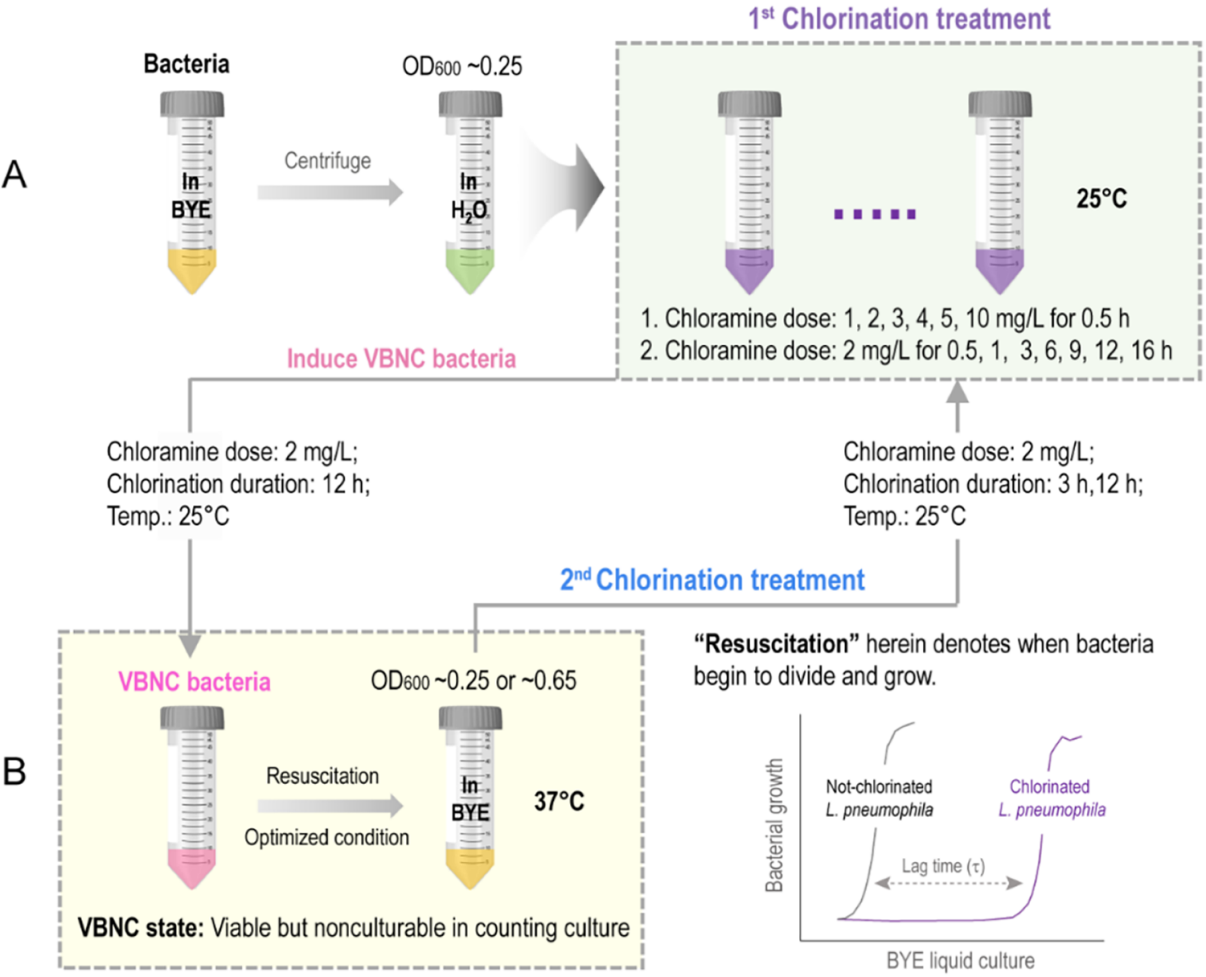
Experimental design. (A) Initial chlorination of *L. pneumophila* was conducted at 25 °C: either different CAT concentrations (0–10 mg/L) for 0.5 h or fixed 2 mg/L CAT for varying durations (0–12 h). This identified 2 mg/L CAT for 12 h as the optimal protocol to generate VBNC cells. (B) VBNC *L. pneumophila* was resuscitated in a BYE liquid culture at 37 °C (BYE-Culture). Stationary phase (OD_600_ ∼ 0.65) and logarithmic-phase (OD_600_ ∼ 0.25) cells underwent a second chlorination (3 or 12 h), followed by subsequent BYE-Culture. Growth behaviors (growth rate (μ) and lag time (τ)) of *L. pneumophila* under no chlorination, starvation, and chlorination were monitored across all groups (lower right corner).

## 2 Materials and Methods

### 2.1 Bacterial Strain and Media Preparation

Pure cultures of *L. pneumophila* ATCC 33152 (Guangdong Microbial Culture Collection Center, Guangzhou, China) were used. Buffered charcoal yeast extract (BCYE) agar plates were prepared per the manufacturer’s instructions; Buffered Yeast Extract (BYE) broth was similarly prepared by omitting charcoal and agar powder. *Legionella* growth promoter (Cat. No. SR0560, Guangdong Huankai Microbial Sci. & Tech Co., Ltd.) was added as a supplement, adjusting the final concentrations of ferric ions and cysteine in the media to 0.25 and 0.4 g/L, respectively. An aqueous solution of chloramine–T trihydrate (CAT, IUPAC name: sodium chloro-(4-methylbenzene-1-sulfonyl)­azanide (3H_2_O), purity 98%, Shanghai Yuanye Bio-Technology Co., Ltd., China) was prepared at 1.6 g/L (active chlorine ∼10%, determined via DPD colorimetric method as total chlorine). Prior to dilution for chlorination, the solution was sterilized through a 0.22 μm filter. *Legionella* culture base (Guangdong Huankai Microbial Sci. & Tech Co., Ltd., China), agar powder (Sigma-Aldrich, MO), and yeast extract (Oxoid Limited, U.K.) were used as received.

### 2.2 Chlorination Treatment of *L. pneumophila* and Post-Treatment Cultivation

To prevent BYE medium from quenching CAT activity (see Supporting Information S2), *L. pneumophila* culture was prepurified in deionized water (DI-H_2_O, pH 6.5). A single colony from BCYE agar was inoculated into 5 mL of BYE medium in a 50 mL centrifuge tube and cultured overnight at 37 ± 0.5 °C with shaking (225 r/min). After dilution in fresh BYE medium (OD_600_ ∼ 0.07) to reach the logarithmic growth phase (OD_600_ ∼ 0.25), the culture was centrifuged at 4800 r/min (4045*g*, TDZ5-WS, Cence Xiangyi, China) for 4–6 min at room temperature to remove the medium. This step was repeated after resuspending the cells in sterile DI-H_2_O, with final OD_600_ adjusted to 0.25 (equivalent to 2.3 ± 0.7 × 10^8^ CFU/mL).

Prepurified cells in DI-H_2_O (0.5 mL) were immediately mixed with 4.5 mL of fresh CAT solution for chlorination (25 ± 0.5 °C, 225 r/min). For each CAT concentration, at least three parallel samples were included as biological replicates (the same for all subsequent experiments). CAT remained stable in DI-H_2_O throughout the experiment (Figure S1A). The use of DI-H_2_O simplified chlorination treatments here, as pH and ionic strength do not significantly alter CAT activity within a broad pH range (5–10).^30^ Tested parameters included CAT concentration (1 to 10 mg/L for 0.5 h) and chlorination duration (0.5 to 16 h at 2 mg/L CAT). The WHO recommends 2–4 mg/L free chlorine for drinking water chlorination, which is significantly higher than the free chlorine content of 0.30 ± 0.03 mg/L in 100 mg/L of CAT (Figure S1B). At designated intervals, samples were briefly centrifuged to remove residual CAT completely (see Supporting Information S3), resuspended in BYE medium, and cultured at 25 ± 0.5 or 37 ± 0.5 °C. Chlorination effects on growth, subpopulation dynamics, and viable counts were analyzed to identify VBNC-inducing conditions (i.e., loss of culturability on BCYE agar but revival under resuscitation conditions). It is worth noting that the residual CAT can be removed via a single brief centrifugation step without significant system perturbation (Supporting Information S3), making sodium thiosulfate for neutralizing residual CAT unnecessary. Furthermore, the subsequent addition of 5 mL BYE medium provides another >10-fold dilution, with barely any residual CAT for the second cultivation.

### 2.3 Growth Behavior in BYE Media

Specific growth rate (μ) and lag time (τ) were evaluated using eq 1,^31,32^ based on optical density values (OD) at 600 nm measured at culture initiation (OD_600___0_) or at subsequent time points (OD_600__*_t_*):

1 $$\mu =\ln\left({\mathrm{OD}}_{600\_t}/{\mathrm{OD}}_{600\_0}\right)/\left(t-\tau \right)$$

where τ corresponds to the *x*-intercept of the ln (OD_600_*t*_/OD_600_0_) vs time plot when the function equals zero. OD_600_ was measured using a Thermo Scientific Multiskan FC plate reader (MA) at 25 °C.

### 2.4 Flow Cytometry (FCM)

Live/dead double staining of *L. pneumophila* was performed using the Backlight LIVE/DEAD Bacterial Viability and Counting Kits (L13152, Invitrogen, CA). The kit contains SYTO 9 and propidium iodide (PI); both were dissolved in sterile DI-H_2_O to prepare working solutions (24 μmol/L SYTO 9, 120 μmol/L PI, stored at −20 °C). Just before staining, chlorinated samples were centrifuged to remove supernatant and then resuspended in DI-H_2_O (10-fold dilution, ∼10^6^ CFU/mL; residual CAT ≤ 0.004 mg/L for 2 mg/L CAT-treated samples). Nonchlorinated samples were prepared and diluted similarly; a subset was heated at 90 °C for 5 min to induce cell death or lethal injury. For staining, 5.0 μL SYTO 9 and 2.5 μL PI working solutions were mixed with 92.5 μL *L. pneumophila* suspension, then incubated for 15 min at 30°C in darkness.

Stained samples were analyzed with a CytoFLEX Flow Cytometer (Beckman Coulter, CA) without washing. Excitation used a 488 nm, 50 mW laser (488/8 nm long-pass filter); SYTO 9 and PI fluorescence signals were collected via 525/40 and 690/50 nm bandpass filters, respectively. Detector gains for forward scatter (FSC), side scatter (SSC), SYTO 9, and PI were set to be 500, 500, 350, and 1500, respectively. A flow rate of 10 μL/min was used to analyze ∼10^4^ cells per measurement; each sample was measured three times as technical replicates consecutively without compensation.

### 2.5 Viable Counting Culture

Chlorinated *L. pneumophila* (2 mg/L CAT, varying durations) was collected by centrifugation after complete removal of residual CAT in supernatant, then resuspended in BYE medium or DI-H_2_O (OD_600_ ∼ 0.25). Aliquots were plated on BCYE agar for CFU enumeration, with incubation at 37 ± 1 °C for 7 days (CFU unchanged over 7 days). Culturable cells were quantified by colony counts. All viability assays were performed in at least triplicate; complete culturability loss was confirmed if no colonies formed from 0.2 mL samples.

### 2.6 Chlorination of Resuscitated *L. pneumophila* Followed by BYE-Culture Again

Chlorinated *L. pneumophila* (2 mg/L CAT, 12 h) was resuscitated in BYE medium at 37 ± 0.5 °C (i.e., BYE-Culture; see Supporting Information S4) to either logarithmic phase (OD_600_ ∼ 0.25) or stationary phase (OD_600_ ∼ 0.65, maintained for 3–4 h). These reactivated cells underwent rechlorination (2 mg/L CAT for 3 or 12 h), followed by BYE-Culture again. Consistent with the first chlorination treatment, the repeat chlorination effects on growth, subpopulation dynamics, and viable counts were analyzed by using identical methods. *L. pneumophila* starved in DI-H_2_O for 12 h was subjected to the same cultivation, harvested in both phases, and then processed identically for comparative analysis.

### 2.7 Statistical Analysis

All data are presented as the mean ± standard deviation (SD) based on at least three independent biological replicates. Paired *t*-tests were performed to compare growth rate differences between control (0 h) and starved bacteria (0.5–3 h). Statistical significance was defined as *p* < 0.05, with *p* values between 0.05 and 0.1 considered indicative of a trend. One-way ANOVA was applied to compare τ among not-chlorinated, regrown (from DI-H_2_O starvation), and resuscitated (from VBNC) groups, with Tukey’s post hoc test used for pairwise comparisons. For other analyses (e.g., subpopulation comparison between original and resuscitated cells), Student’s *t*-tests were performed with Bonferroni correction for multiple pairwise comparisons. Due to duplicated pairwise comparisons, the significance threshold was adjusted from *p* < 0.05 to *p* < 0.025 or 0.0125 as appropriate.

## 3 Results

### 3.1 Subpopulation Differentiation under Prolonged Low-Dose Chlorination Differs from Starvation

Similar to chlorinated *E. coli* ^33^ and nutrient-starved/heat-treated *L. pneumophila*,^5,6^ low-dose chlorinated *L. pneumophila* displayed subpopulation variations along a triphasic trajectory in a “crescent moon” pattern, as shown in two-dimensional SYTO 9/PI scatter plots (Figure 2). This pattern reflects a shift from cells with intact membranes (strong green/weak red fluorescence) to those with severely compromised membranes (increased red/diminished green fluorescence)^34^—a universal transition during bacterial inactivation, evidenced by similar patterns in both exponential- and stationary-phase cells (Figure 2C). Subpopulations were thus classified as gate P1 (actively growing cells); gate P3 (dead or significantly damaged cells, validated by exclusive P3 localization of heat-treated cells [95 °C, 5 min], rightmost panel in Figure 2C); and gate P2 (intermediate state between P1 and P3). This framework aligns with established classifications.^33,35^ The P1/P2 boundary was defined using log-phase subpopulation segregation (leftmost panel in Figure 2C), consistent with that of 1 h of H_2_O-starved bacteria (Figure 2A). Notably, the P2 subpopulation represents a heterogeneous cell mixture, with its composition modulated by chlorination dose and duration.^36^

**Figure 2:**
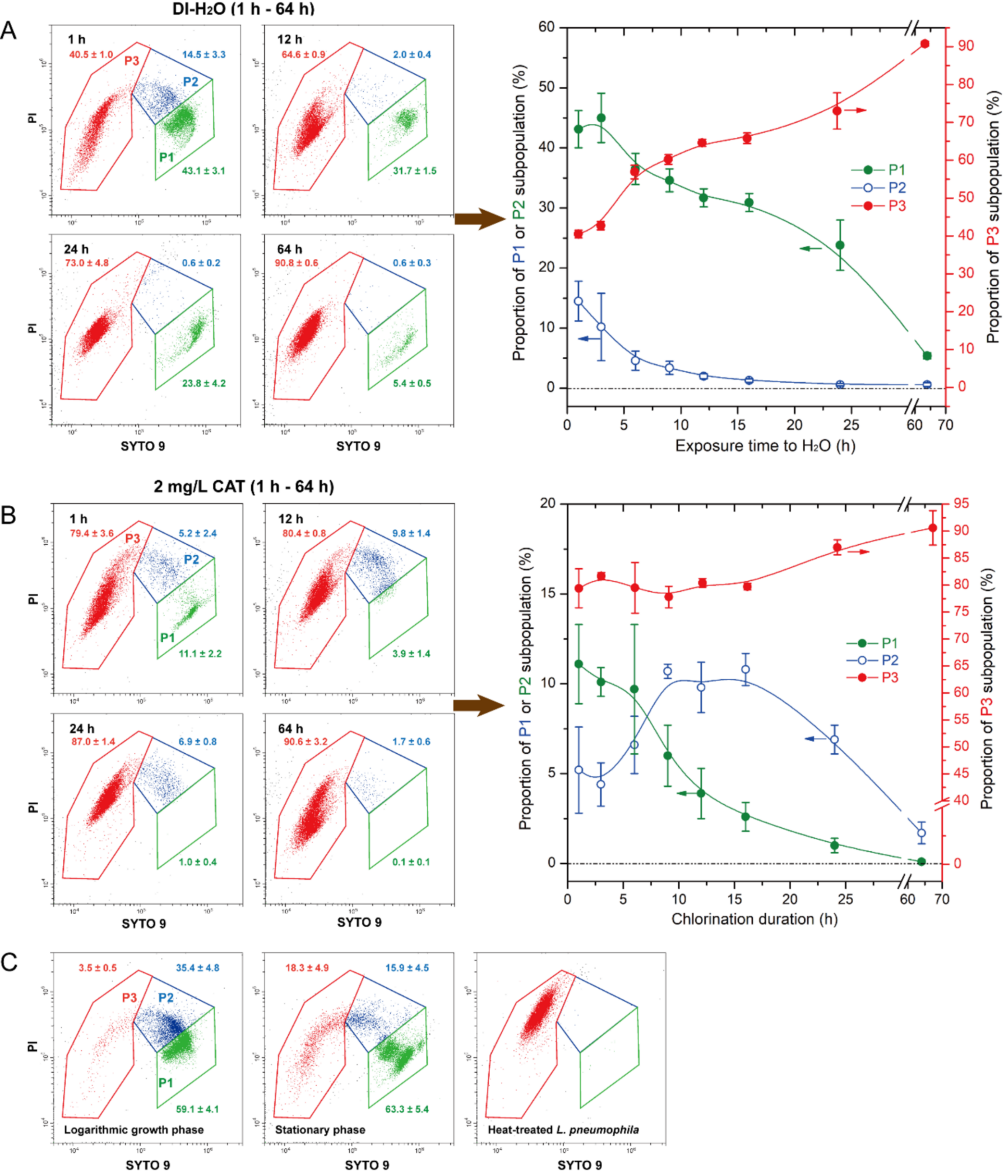
Distinct subpopulation development under starvation or chlorination. (A, B) Representative FCM scatter plots of *L. pneumophila* exposed to DI-H_2_O (A) or 2 mg/L CAT (B) for 1–64 h at 25 °C; temporal trends in subpopulation distributions are plotted to the right. (C) Untreated *L. pneumophila* (logarithmic/stationary phases) and heat-treated cells (95 °C, 5 min) are compared with starved (A) or chlorinated (B) populations.

Untreated *L. pneumophila* (log- and stationary-phase) predominantly consisted of P1 (59.1%–63.3%) with minimal P3 (Figure 2C). Exposure to low-dose chlorination (2 mg/L CAT, 1–64 h) induced progressive subpopulation redistribution: the P3 proportion in *L. pneumophila* increased from ∼80% for 1 h of chlorination to ∼90% following 64 h of treatment (right panel of Figure 2B). A critical window emerged at 9–16 h of chlorination, where P2 abundance peaked at 9.8% ± 1.4%—in sharp contrast to H_2_O-treated cells, which showed progressive P2 depletion from 14.5% ± 3.3% (1 h) to 1.3% ± 0.3% (≥ 16 h) (right panel of Figure 2A). While nutrient-free DI-H_2_O starvation gradually increased the P3 subpopulation from 40% (1 h) to 90% over 64 h, chlorination achieved comparable lethality within 1 h, highlighting its rapid bactericidal effect. Diminished SYTO 9/PI fluorescence in the P3 subpopulation of treated *L. pneumophila* (vs heat-treated counterparts) suggests progressive nucleic acid degradation for both stressors, chlorination and starvation.^5,37^

### 3.2 VBNC Induction and Resuscitation Dynamics following 12 h Low-Dose Chlorination

These once-chlorinated cells were cultured in liquid BYE medium (i.e., BYE-Culture) and on solid BCYE agar to assess growth behavior. The longer the 2 mg/L CAT treatment is, the greater the lag in *L. pneumophila* regrowth in BYE-Culture is (Figure 3A). As expected, cells chlorinated for >12 h hardly grew on BCYE agar even after 7 days, confirming their “non-culturable” status (Figure 3B). Nevertheless, 12 h-chlorinated bacteria revived in BYE liquid medium, albeit with a ∼10-fold longer lag time (∼47 h) vs the ∼4–6 h lag time for *L. pneumophila* exposed to 2 mg/L CAT (0 h) momentarily (just enough time to put the cells in, then wash immediately) and those treated by DI-H_2_O (0 mg/L CAT) for 0–12 h. This result aligns with its subpopulation profile: ∼4% P1, ∼10% P2, and ∼80% P3 (left panel of Figure 2B). For 16 h-chlorinated bacteria, however, only 3 out of 9 samples were revived under the same conditions, with an even more extended lag time (∼54 h). In parallel, *L. pneumophila* exposed to DI-H_2_O for 12 and 16 h grew readily on BCYE agar (both “culturable”; Figure 3B), consistent with their high P1 abundance (∼30%) (left panel of Figure 2A). Thus, unlike water starvation, 12 h low-dose chlorination induces a VBNC state to promote cellular adaptation over cell death.^38^ Resuscitation of VBNC cells thus requires not only nutrient-rich liquid media but also incurs significant lag time, reflecting metabolic reactivation barriers.

**Figure 3:**
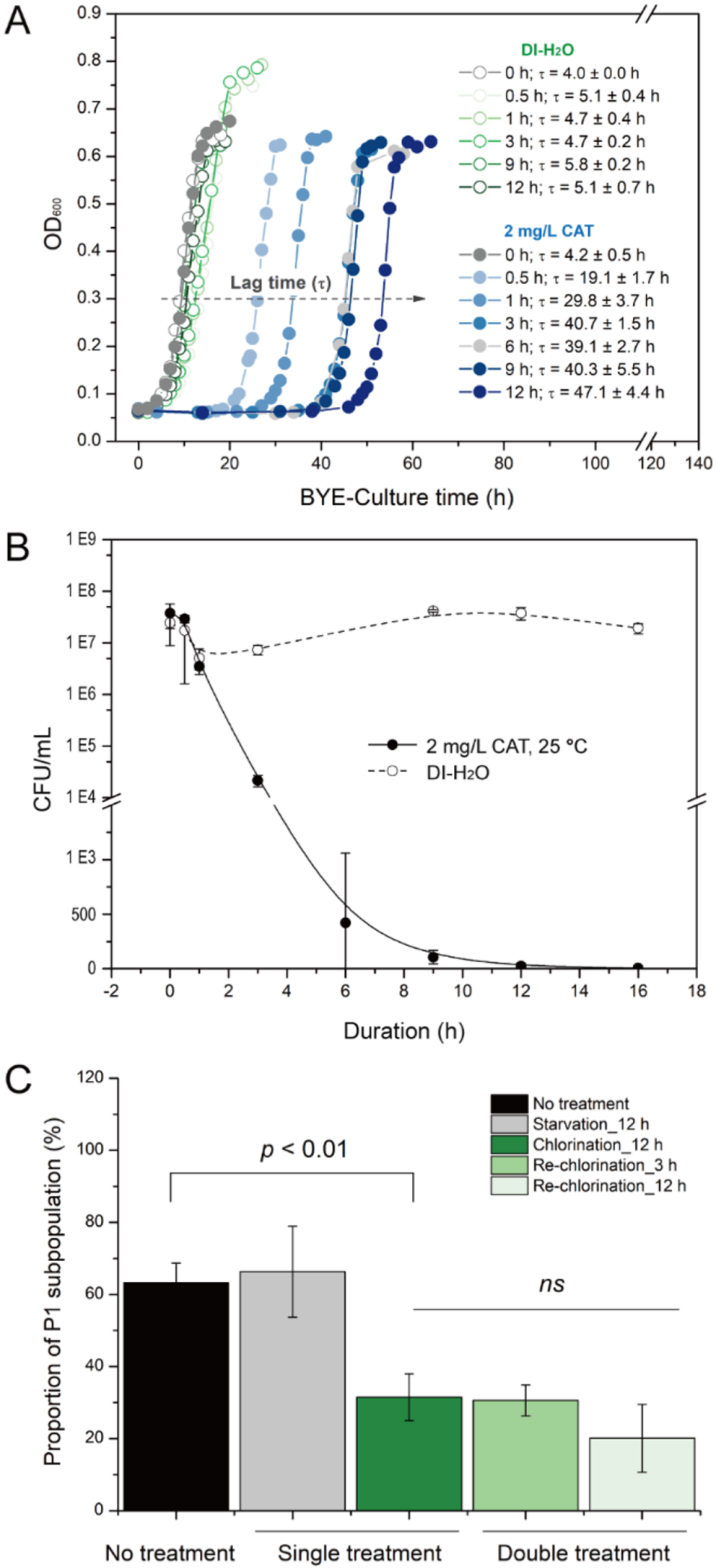
(A, B) Growth of *L. pneumophila* after starvation (DI-H_2_O) and chlorination. (A) Growth curves in BYE-culture after starvation (DI-H_2_O, open circles) or chlorination (2 mg/L CAT, solid circles) for 0–12 h. (B) Viable counts vs duration for starvation (DI-H_2_O, open circles) or chlorination (2 mg/L CAT, solid circles, fitted line). Note: lag time τ increases with chlorination duration but remains stable under starvation. (C) Proportion of P1 subpopulation in differently treated cells: 12 h starvation in DI-H_2_O or 12 h single chlorination (2 mg/L CAT)—these two groups of treated cells were subjected to BYE-Culture, then harvested at the stationary phases (both vs not-treated parent *L. pneumophila*)—and rechlorination (2 mg/L CAT, 3 h/12 h) was performed on the stationary-phase resuscitated cells. *ns*: no significant difference (tested by Student’s *t*-test, before vs after treatment).

### 3.3 Chlorine Tolerance Acquisition by Resuscitated VBNC *L. pneumophila*

*L. pneumophila* revived from the VBNC state after 12 h of chlorination exhibited markedly different subpopulation profiles compared to untreated parent cells. During the logarithmic growth phase, resuscitated bacteria showed 40.1% ± 5.5% P1 and 45.9% ± 9.9% P2, shifting from the original 59.1% ± 4.1% P1 and 35.4% ± 4.8% P2 in untreated cultures (Figure 2C; *p* < 0.02 for P1; *p* > 0.2 for P2). In stationary phase, revived cells maintained 31.5% ± 6.5% P1 and 40.1% ± 18.5% P2, contrasting with 63.3% ± 5.4% P1 and 15.9% ± 4.5% P2 in parent cells (Figure 3C; *p* < 0.01 for P1; *p* > 0.15 for P2). Notably, *L. pneumophila* regrown after 12 h starvation in DI-H_2_O maintained subpopulations comparable to untreated parent cells (all *p* > 0.4), with 49.9% ± 15% P1 and 41.0% ± 15.3% P2 during log phase, and 66.3% ± 12.6% P1 and 15.0% ± 5.7% P2 in stationary phase. Further, FCM analysis revealed these distinct subpopulation compositions reflect fundamental cellular adaptation triggered by prolonged low-dose chlorination through VBNC induction.

Subsequent experiments subjected resuscitated *L. pneumophila* to repeat chlorination followed by post-treatment cultivation (Figure 1B). Here, “twice-chlorinated *L. pneumophila*” denotes resuscitated VBNC cells re-exposed to identical chlorination conditions for varying durations, while “starvation-chlorinated *L. pneumophila*” refers to cells starved in DI-H_2_O for 12 h, regrown, and then subjected to chlorination for a second time under the same protocol. When rechlorination during the logarithmic growth phase, both groups displayed subpopulation profiles analogous to their once-chlorinated counterparts at 3 and 12 h (Figure S4). However, differential responses emerged in stationary-phase cells. Twice-chlorinated *L. pneumophila* retained 30.6% ± 4.3% (3 h) and 20.1% ± 9.4% (12 h) of P1 subpopulations (Figure 4A), corresponding to levels equivalent to and slightly lower than pretreatment values (31.5% ± 6.5% P1, Figure 3C), respectively. In contrast, starvation-chlorinated cells retained half (31.5% ± 1.0% P1) and one-seventh (9.5% ± 4.6% P1) of the initial P1 subpopulation (66.3% ± 12.6%, both *p* < 0.05) at 3 and 12 h, respectively (Figure 4B). These results differ markedly from single-chlorination outcomes, where residual P1 percentages significantly decreased to ∼10% (3 h, one-sixth) and ∼4% (12 h, one-16th) of untreated levels (Figure 4A-1,B-1). This coordinated response—sustained viability (P1, Figure 3C) relative to starvation-chlorinated and single-chlorinated cells—supports the conclusion that VBNC-derived *L. pneumophila* become more chlorine-tolerant postresuscitation, particularly in 3 h (vs 12 h) repeat chlorination challenges, via prolonged sublethal chlorine exposure, more effective than starvation stress alone.

**Figure 4:**
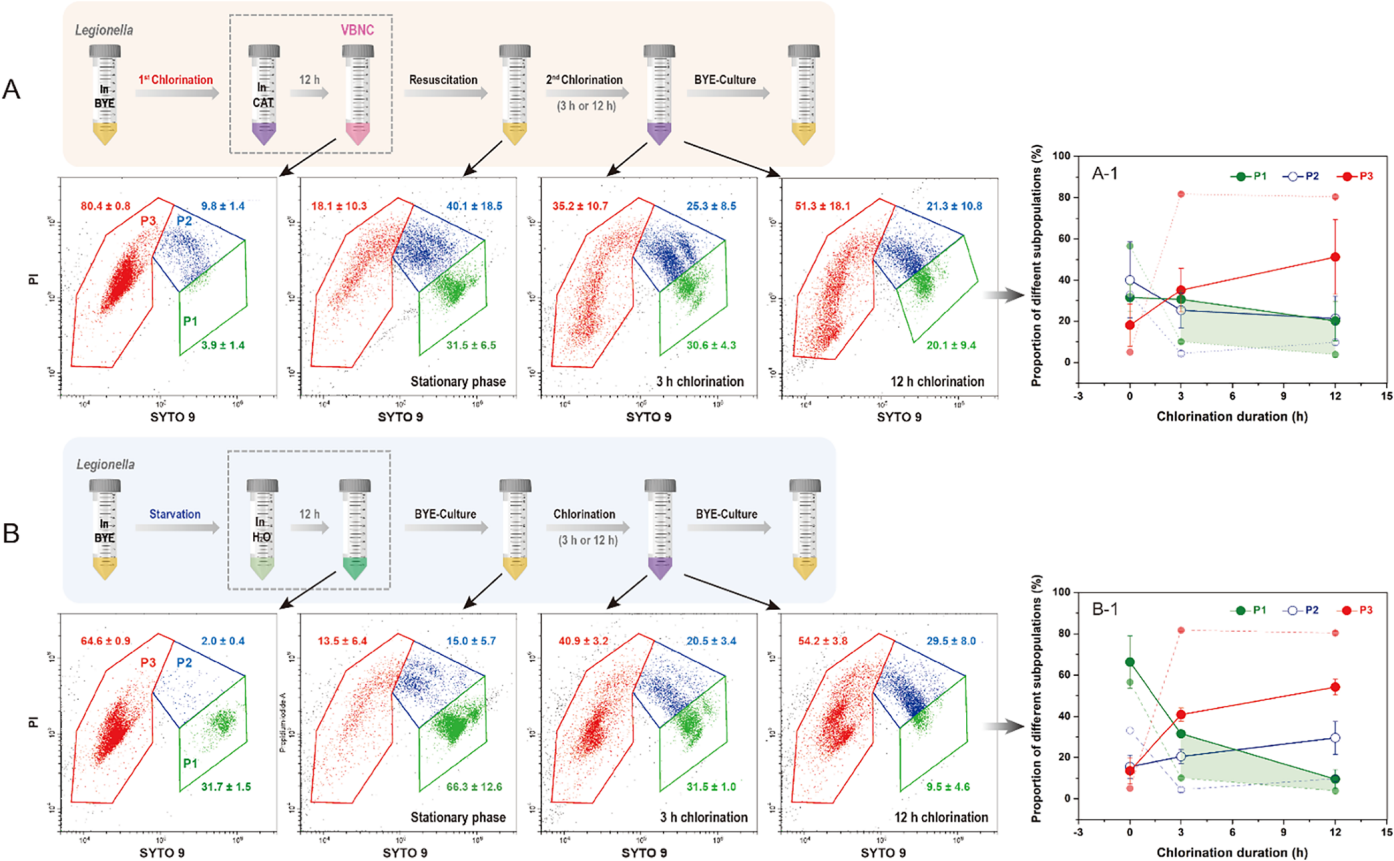
Subpopulation dynamics of *L. pneumophila* under sequential treatments. (A) First chlorination (2 mg/L CAT, 12 h), followed by resuscitation and repeat chlorination (3 or 12 h). (B) Parallel workflow substituting chlorination with 12 h starvation in DI-H_2_O. Representative FCM scatter plots (left to right) show subpopulations at key stages: post-treatment (chlorination/starvation), stationary-phase resuscitation harvest, and after rechlorination/chlorination (3 or 12 h). (A-1, B-1) Compared with single-chlorinated *L. pneumophila* (dashed-line trends replotted from Figure 2B), proportions of P1 and P2 subpopulations remained high after 3 h treatment in both double-chlorination and starvation-chlorination groups, with concurrent reduction in P3. Notably, P1 declined significantly following 12 h of chlorination in the starvation-chlorination group.

### 3.4 Enhanced Tolerance under Repeat Chlorination Treatment

Further evidence that VBNC *L. pneumophila* constitutes a crucial step in chlorine tolerance development was observed in growth dynamics during the second postchlorination BYE-Culture. While repeat chlorination during the logarithmic growth phase showed no significant growth variations (Table S4), twice-chlorinated *L. pneumophila* “woke up” more readily than once-chlorinated cells when the second treatment targeted stationary phase populations (Figure 5A). Following 3 and 12 h of repeat chlorination, the lag times (τ) of twice-chlorinated *L. pneumophila* were 26.8 ± 3.0 h and 41.2 ± 0.8 h, respectively: the 3 h group had a markedly shorter lag time than once-chlorinated cells from the first postchlorination BYE-Culture (40.7 ± 1.5 h), while the 12 h group trended toward shorter lag times (vs 47.7 ± 4.0 h, *p* = 0.08). In stark contrast, starvation-chlorinated *L. pneumophila* maintained a similar τ (∼40.6 ± 6.0 h) across both durations—no statistical difference from single chlorination for 3 h, but a slightly shortened lag time at 12 h (*p* < 0.01). In these experiments, all groups had nearly identical growth rates (μ ≈ 0.3 h^–1^), except short-term (0.5–3 h) starved bacteria, which showed distinct growth rates vs control (0 h) (Figure 5B). See Supporting Information S5 for further analysis.

**Figure 5:**
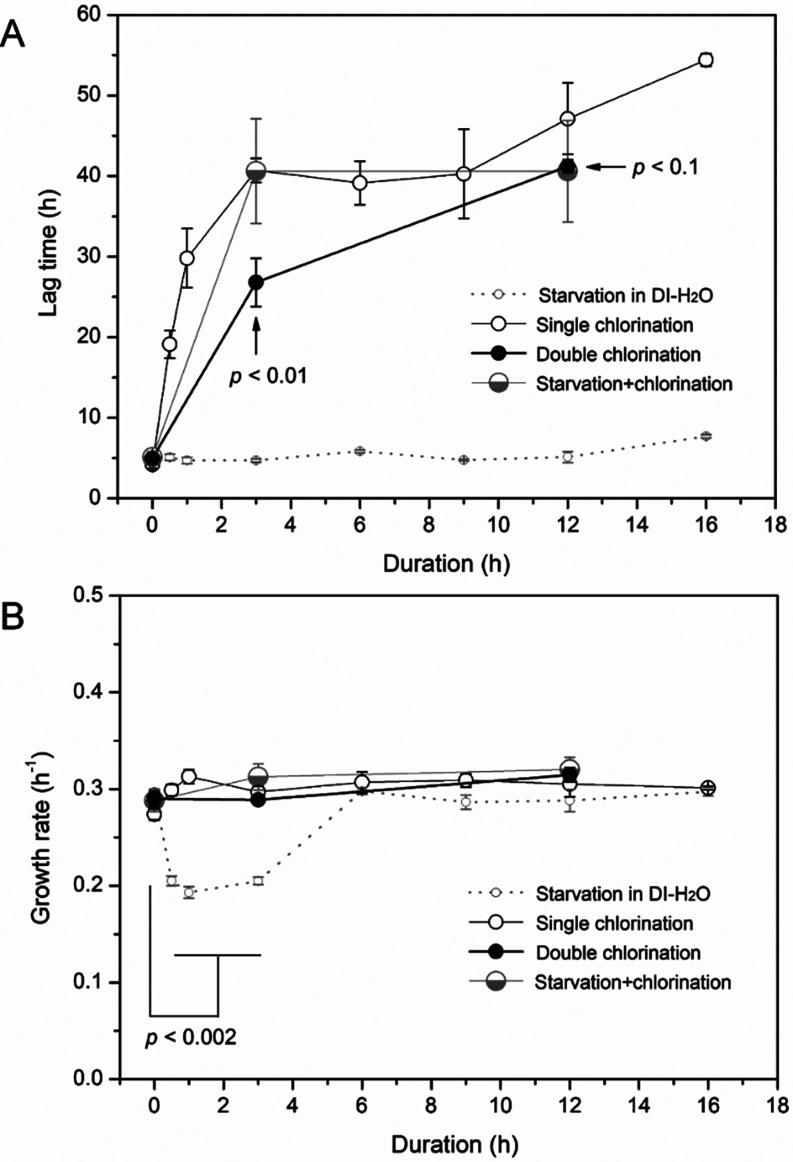
Growth behavior characterization. Lag times (A) and growth rates (B) of *L. pneumophila* in BYE-Culture after four treatments with varying durations. Data for the first two treatments (small gray open circle: starvation in DI-H_2_O; medium black open circle: single 2 mg/L CAT treatment) are from Figure 3A. The third and fourth treatments correspond to those in Figure 4A,B, respectively. Stationary-phase resuscitated *L. pneumophila* (medium black closed circle) and cells recovered from 12 h DI-H_2_O starvation (large gray half-open circle) were each subjected to 2 mg/L CAT treatment for 3 and 12 h. *p* values: twice-chlorinated vs single-chlorinated cells (A); starved cells for 0.5 h–3 h vs control (0 h) (B).

Combined with the above FCM results, the shorter lag time of twice-chlorinated *L. pneumophila*—vs single-chlorinated and starvation-chlorinated cells (subjected to identical chlorination) likely stems from subpopulation profile variations in VBNC-revived *L. pneumophila*. These variations that reflect complex adaptation strategies by the cells to environmental stressors enhanced chlorine tolerance, thereby affecting lag time without altering growth rate.

## 4 Discussion

### 4.1 VBNC-Mediated Adaptation Promotes Chlorine Tolerance

Our findings provide experimental evidence directly linking VBNC transformation to chlorine tolerance development, which aligns with the recent work by Daer et al.^39^ In their 24-cycle adaptation experiments, they observed that 2.5 mg Cl_2_/L sodium hypochlorite induced *E. coli* into VBNC states, and these cells subsequently exhibited significant chlorine resistance. Besides chlorine as a stressor, Yang et al.^40^ reported rapid phenotypic adaptation in *E. coli* via intermittent ampicillin exposure cycles interspersed with antibiotic-free recovery—a progression mirroring chlorine resistance development. Notably, CRB are also more likely to be antibiotic-resistant.^41^ Though mechanisms differ, both disinfectants and antibiotics drive bacterial selection; this parallelism suggests that antibiotic resistance research may inform chlorine resistance studies, given shared evolutionary trajectories potentially involving overlapping molecular pathways. Intermittent antibiotic exposure accelerates resistance evolution via tolerance mechanisms that elevate viable cell proportions and mutation opportunities.^42,43^ Collectively, these data underscore the VBNC transition as a critical adaptive mechanism in bacterial chlorine resistance acquisition.

Transition to the VBNC state relies on two core systems: RpoS (sigma factor)-mediated stress response (controlling stress adaptation programs) and (p)­ppGpp-driven stringent response (activating toxin-antitoxin (TA) systems to arrest growth).^14,15,44^ During dormancy establishment, (p)­ppGpp coordinates with RpoS to synergistically induce morphological/metabolic remodeling and virulence regulation under stress,^45^ ensuring long-term survival.^46^ For example, in chlorination-induced VBNC *E. coli*, elevated RpoS triggers dual defenses: *soxR*/*katG*-mediated oxidative resistance^47^ and efflux pumps/porins upregulation for toxin clearance.^8,9^ Moreover, the distinct lag time variations between log-phase and stationary-phase cultures during repeated chlorination treatments (Table S4) imply acquired chlorine tolerance may involve stationary phase-associated epigenetic adaptations regulated by the stringent response.^48^ For example, *L. pneumophila* in the stationary phase upregulates LD-transpeptidase levels, remodeling peptidoglycan structure to thicken the protective cell envelope.^49,50^ Nevertheless, the precise VBNC entry mechanism remains unknown, with no defined universal mechanism.^51^

Future research to close the critical knowledge gaps in the environmental sensing mechanisms preceding resuscitation is warranted, because revival stimuli do not consistently succeed to revive VBNC cells;^13,52,53^ this is much needed to advance the understanding of resistance development. Current models propose that partial breakdown of cell wall peptidoglycan may evoke VBNC resuscitation, directly or indirectly (e.g., released small muropeptides acting as “second messengers”),^13,54^ potentially via RpoS-mediated reactivation.^55^ Breakthrough work identifies ATP-dependent protein aggregation as a dormancy depth indicator,^56^ providing a pathway to streamline identification of critical regulatory mechanisms. Addressing these knowledge gaps requires further research on the VBNC state to unravel the mechanisms underlying chlorine resistance development.

### 4.2 Lag Time Reflects the Repair Capacity of Chlorine-Damaged Cells

Multiple methodologies, including the minimal inhibitory concentration (MIC) assay, have been used to assess bacterial chlorine resistance and/or tolerance,^19^ but these methods provide limited mechanistic insight into resistance development. A historical approach for monitoring bacterial chlorine resistance is repeated chlorination treatment, though early studies failed to reach consistent conclusions.^57,58^ Leyval et al.^59^ attributed such discrepancy to different experimental protocols, specifically the use of different progeny for rechlorination. Gao and Liu^60^ treated nine *Listeria monocytogenes* strains through 10–20 chlorination cycles at increasing concentrations of CAT and sodium hypochlorite (from 0.5 × MIC to MIC). Adapted strains grew stably in the same medium with these disinfectants at MIC; i.e., they became CRB. Based on these findings, this study further elucidates the biological basis of such chlorine resistance evolution in *L. pneumophila*. In particular, the growth dynamics, especially the lag time, is interpreted to indicate damage repair capacity of the chlorine-exposed cells, and is discussed below.

Our experiments targeted early-stage VBNC cells. Although these cells share molecular characteristics with persisters, which resume growth quickly on standard culture medium once stressors are removed,^61^ VBNC cells specifically refer to those in relatively deeper dormancy.^4,44,62^ Because chlorinated cells were unable to grow on nutrient agar (Figure 3B), making them distinct from persister cells, and were able to grow in nutrient-rich liquid medium even without specific stimuli (e.g., amino acids, pyruvate, or glutamate),^47,63,64^ we interpret this to mean these early-stage VBNC cells acquired only minimal dormancy, compared with late-stage VBNC cells that may be even more dormant.

Our study finds that lag time τ emerges as a critical parameter to evaluate the state of dormancy (Figure 3A): Extended τ values may indicate severe chlorine-induced cellular damage, necessitating prolonged repair periods, from membrane reconstruction to metabolic restoration.^65,66^ FCM results validate this correlation, demonstrating that shorter lag times correlate with higher proportions of active cells (P1 subpopulation) and reduced dead-cell-analogous fractions (P3 subpopulation) compared with pretreatment conditions (Figure 4). Consequently, the decreased τ value after repeated chlorination treatments indicates adaptive evolution in these minimally dormant VBNC cells, confirming acquired chlorine tolerance (Figure 5).

Although methodological refinements (e.g., extending chlorination duration to 14 h or implementing single-cell analysis) could further validate VBNC status, multiple lines of evidence indicate that *L. pneumophila* revived after 12 h of chlorination (2 mg/L CAT) originates from VBNC cells rather than residual survivors. Residual survivors would be expected to exhibit subpopulation profiles similar to untreated parent cells during resuscitation, as seen in regrown cells following 12 h starvation in DI-H_2_O. This characteristic subpopulation pattern, however, is not observed in chlorination-revived *L. pneumophila*, particularly in stationary-phase cultures, distinguishing them from both not-treated parent cells and starvation-regrown cells (Figures 2, 4 and S4). How such an augmented chlorine tolerance in *Legionella* may impact its infectivity toward macrophages and amoebae is another topic worthy of further investigation.

## 5 Conclusions

Our findings reveal that chlorination-induced VBNC or minimally dormant *L. pneumophila* exhibit enhanced chlorine tolerance upon resuscitation, particularly those harvested in the stationary phase, with epigenetic reprogramming (rather than permanent genetic changes) as the likely mechanism. Combined with existing evidence, it is likely that such tolerant cells could further evolve into CRB. This study thus firmly establishes a VBNC-mediated link between chlorination stress and the emergence of bacterial chlorine tolerance, offering a novel target for decoding resistance evolution in waterborne pathogens. Further studies addressing knowledge gaps in VBNC induction/resuscitation mechanisms will deepen our understanding of VBNC-mediated adaptation and inform disinfection practices.

## Supplementary Material



## Notes

Nutrient and temperature impacts on *L. pneumophila* growth, long-term free chlorine stability in DI-H_2_O (Figure S1); CAT removal by centrifugation (Figure S2); chlorination treatment optimization (Figure S3), and DI-H_2_O exposure impacts on *L. pneumophila* growth; *L. pneumophila* subpopulation proportion changes during the chlorination of resuscitated log-phase cells (Figure S4); summarize *L. pneumophila* growth dynamics under various conditions (Tables S1–S4) (PDF)
