# Supplementary material for: Repeat Prolonged Chlorination at Low Dose Induces Chlorine Tolerance in Legionella pneumophila via Viable but Non-culturable State
Source: Environ Health (Wash). 2025 Oct 6;4(2):291–300. doi: 10.1021/envhealth.5c00360 (PMC12930306; doi:10.1021/envhealth.5c00360)
Supplement: Supplementary file 1 [file eh5c00360_si_001.pdf]

Supporting Information

Repeat Prolonged Chlorination at Low Dose Induces Chlorine Tolerance in *Legionella pneumophila* via Viable but Non-culturable State

Xiaofei Yuan[†,‡,§] and Yan Zheng[*,†,§]

[†] State Key Laboratory of Soil Pollution Control and Safety, School of Environmental Science and Engineering, Southern University of Science and Technology, Shenzhen 518055, China
[‡] Research Centre for Life Science Computing, Zhejiang Laboratory, Hangzhou 311100, China
[§] Guangdong Provincial Key Laboratory of Soil and Groundwater Pollution Control, School of Environmental Science and Engineering, Southern University of Science and Technology, Shenzhen 518055, China

*Email: yan.zheng@sustech.edu.cn.

This file includes:

S1: Impact of nutrients and temperature on the growth of *L. pneumophila*

S2: Stability of CAT in DI-H_2O

S3: Removal of CAT by centrifugation

S4: Optimization of chlorination treatment

S5: Impact of starvation in DI-H_2O on bacterial growth

Figure S1: Abundance of free chlorine in CAT solutions and the stability of CAT in DI-H_2O

Figure S2: Decrease in total and free chlorine amounts after each centrifugation.

Figure S3: Impact of chlorination on *L. pneumophila.*

Figure S4: Alterations in the proportion of *L. pneumophila* subpopulations.

Tables S1 to S4: Growth dynamics of *L. pneumophila* under various conditions.

S1 : Impact of nutrients and temperature on the growth of *L. pneumophila*

L. pneumophila is an auxotroph that favors amino acids as carbon and energy sources [1-3]. In BYE medium, yeast extract serves as the sole nutrient source for *Legionella*. No bacterial growth occurred when yeast extract was omitted, whether incubated at 25°C for 168 h or 37°C for 59 h. Subsequently, a single colony was inoculated into 5 mL of BYE medium containing 10%, 50%, or 100% of the original yeast extract concentration. Cultures were incubated at 25 ± 0.5°C or 37 ± 0.5°C with shaking (225 r/min). The growth rate (μ) and lag time (τ) were calculated from OD_{600} measurements (see Methods).

At 37°C, *L. pneumophila* exhibited exponential growth for 5-7 h in nutrient-rich BYE media (≥ 50% yeast extract), with doubling times of 2.2 ± 0.1 h or 2.8 ± 0.3 h, consistent with prior findings [4]. Elevated temperature accelerated growth, particularly in nutrient-rich conditions: μ tripled when temperature increased from 25°C to 37°C (Table S1). Similarly, increasing yeast extract from 10% to 100% raised μ by 4-fold at 25°C and 9-fold at 37°C. Lag time τ halved in nutrient-rich media with higher temperatures, though minimal differences were observed at 10% yeast extract. Notably, τ in BYE medium with 100% yeast extract exceeded values in lower-nutrient conditions at all temperatures, potentially due to "nutrient shock" [5] from colony inoculation (versus liquid culture). This parallels the use of low-nutrient R2A agar for enriching environmental bacteria [6]. Collectively, abundant nutrients and optimal temperatures (i.e. 32–37°C [2, 7]) promote *L. pneumophila* growth.

S2: CAT demonstrates long-term stability in DI-H_2O

Consistent with previous reports [8], when CAT is dissolved in yeast extract-free BYE medium at 8.5 mg/mL, no detectable active chlorine is observed, likely due to quenching by organic material in the medium. To evaluate its stability in DI-H_2O, a fresh CAT solution (8 mg/mL) was diluted to 200 mg/L and stored under chlorination conditions (e.g., in a 50 mL centrifuge tube with shaking at 25 °C) for 24–48 h. Periodically, aliquots were 10-fold diluted in chlorine-demand-free container, and free chlorine was measured using a HACH DR300 meter. Triplicate measurements showed a slight decline in free chlorine levels over time, though dilution-related variability was observed (Figure S1A). These results indicate negligible chlorine demand in DI-H_2O, confirming CAT's suitability as a disinfectant for long-term chlorination (≤ 48 h). Figure S1B illustrates free chlorine levels across CAT concentrations.

S3: CAT removal by centrifugation

To eliminate residual CAT after postchlorination, 5 mL of 15.2 mg/mL CAT was centrifuged (4,800 r/min, 6 min). After discarding 4.5 mL of supernatant, an equal volume of DI-H_2O was added, and centrifugation was repeated until free chlorine became undetectable in the supernatant. Total and free chlorine decreased linearly per centrifugation cycle (~10-fold dilution per step; Figure S2). Given the low CAT concentration (2 mg/L) during chlorination and the quenching effect of BYE medium (refer to Section S2), only a single centrifugation

step was applied, where the supernatant was completely discarded before resuspending the pellets in BYE medium for subsequent culturing.

S4: Optimization of chlorination treatment

At 0.5 h chlorination, 5 mg/L CAT marked a threshold: lower concentrations allowed regrowth in BYE medium at 37°C (termed BYE-Culture, see Section S1), while 10 mg/L CAT completely inhibited regrowth in BYE-Culture within 88 h (Figure S3A). Lag time (τ) increased proportionally with CAT concentration, while growth rate (μ slightly rose until 2 mg/L CAT (Table S2). At fixed CAT concentration (2 mg/L), τ increased linearly with treatment duration (0–3 h), plateauing thereafter (Figure 5A, Table S3). μ remained stable (Figure 5B and Table S3). After 16 h chlorination, 3/9 samples regrew in BYE-Culture within 172 h (μ = 0.301 ± 0.002 h^{-1}, τ = 54.4 ± 0.8 h). In contrast, DI-H_2O-exposed cells showed constant τ (4–5 h). Short treatments (≤ 3 h) more drastically reduced μ (discussed in Section S5). Based on regrowth kinetics, 2 mg/L CAT was selected for subsequent experiments.

Lowering BYE-Culture temperature from 37°C to 25°C increased τ independently of chlorination duration (Figure S3B). Chlorinated *L. pneumophila* (2 mg/L CAT, 6 h) failed to regrow in DI-H_2O or yeast extract-free BYE medium (37°C, 130 h), but recovered in 10% yeast extract BYE medium with significant changes in both μ and τ (Figure S3C, Table S2). Nutrient-rich conditions and elevated temperatures are thus critical for repairing chlorination-induced damage. Prolonged chlorination (> 16 h) or high CAT concentration (10 mg/L) likely caused irreversible damage; detailed μ and τ values are summarized in Tables S2 and S3.

In contrast to DI-H_2O exposure, prolonged chlorination reduced CFU linearly over 0.5 h, plateauing near zero after 12 h (Figure 3B). Lag time τ corelated with CFU, stabilizing around 40 h for < 10^4 CFU/mL (Figure S3D). FCM revealed 10% viability (P1 subpopulation) after 3 h chlorination (2 mg/L CAT; Figure 2B), suggesting a viability threshold below which τ is no longer determined by viable cells [9]. Extended τ after 12 h chlorination (≥ 47 h) likely reflects reduced resuscitation capability of VBNC bacteria with increasing chlorination severity. These findings align with FCM data (Figure 2B) and prior reports on VBNC state induction [10-11].

S5: Impact of starvation in DI-H_2O on bacterial growth

Exposure to DI-H_2O stresses *L. pneumophila* due to the absence of essential nutrients and growth promotors, such as L-cysteine and ferric pyrophosphate[1-3]. Notably, *L. pneumophila* exhibited an immediate response to nutrient deprivation, with growth rates sharply declining even under optimal temperature conditions within the initial hours of treatment (Figure 5B and Table S3). However, μ recovered when exposure exceeded 3 h. This aligns with findings by Li et al.[12], who observed rapid transcriptomic adaptations in *L. pneumophila* within 2 h of exposure to a nutrient-poor artificial freshwater medium. The transient growth suppression —characterized by reduced growth rates but stable lag times (Figure 5A) —likely reflects a stringent response of *L. pneumophila* prior to full adaption, which stabilizes after ~6 h. Despite this, the bacteria retained growth activity for up to 16 h, consistent with prior reports [12-13] but deviating from the monotonous increase in the P3 subpopulation

(4%–66% viability loss by FCM; Figure 2A). This divergence may arise from methodological differences in viability assessment [14], may also be linked to the subpopulation classification criteria. Prolonged DI-H_2O exposure (64 h) further reduced τ (> 122 h) and μ (0.076 h^{-1}), corroborating the mortality trend in Figure 2A. Thus, within our experimental framework, DI-H_2O exposure alone did not significantly amplify or distinctively contribute to chlorination-induced effects on *L. pneumophila*; its impact may have been obscured by concurrent stressors.

Supplemental Figures

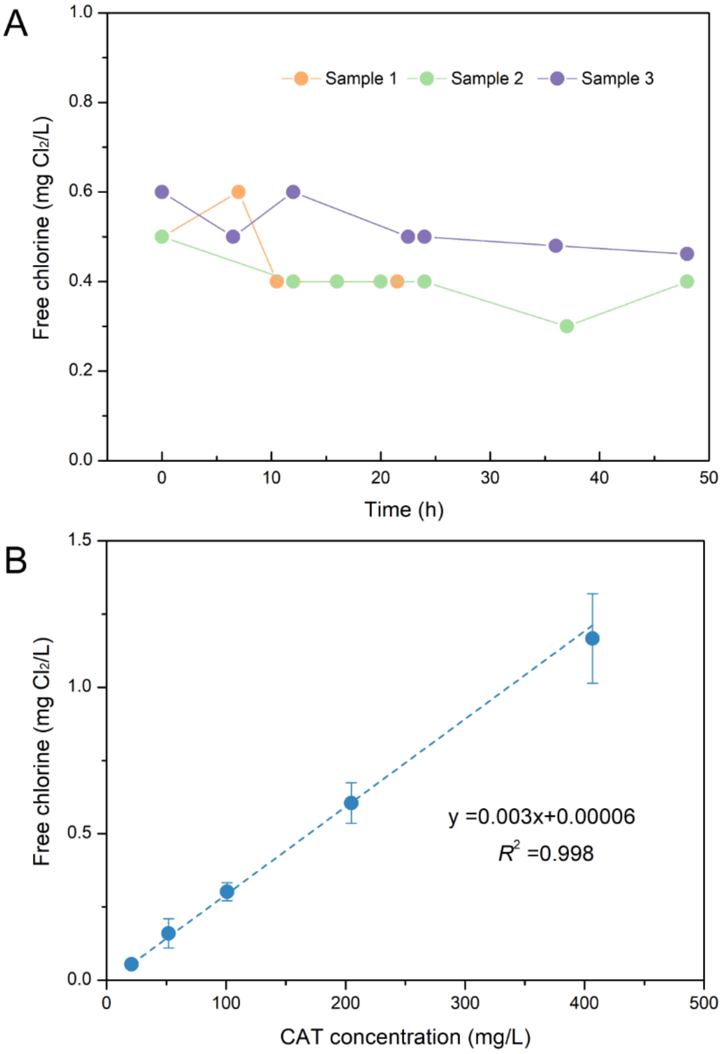

Figure S1. Free chlorine levels and CAT stability in DI-H_2O. (A) Temporal variation of free chlorine concentration in a 204 mg/L CAT solution at 25°C. (B) Correlation between free chlorine concentration and initial CAT dose in freshly prepared solutions.

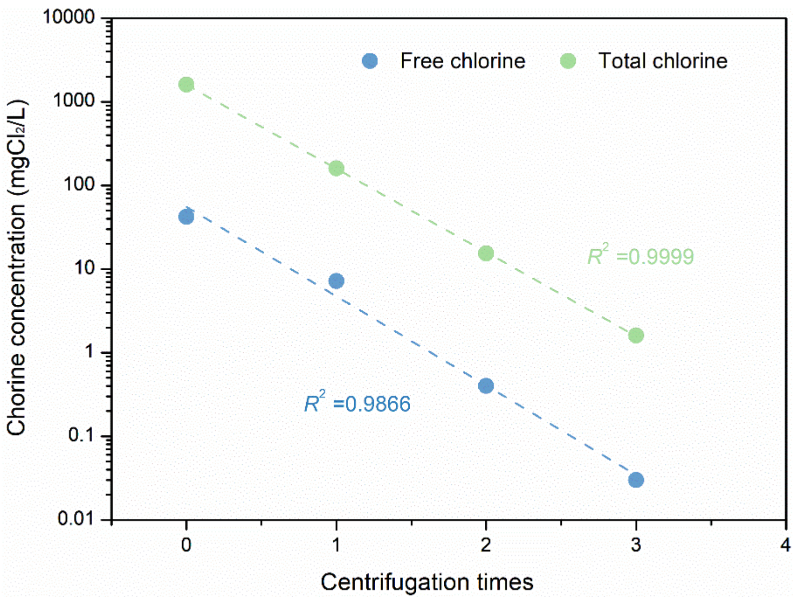

Figure S2. Reduction of free and total chlorine by centrifugation. Decline in the supernatant chlorine concentrations after sequential centrifugation steps (4,045 × g, 6 min). Dashed lines indicate linear regression fits (R^2 = 0.9999 and 0.9866).

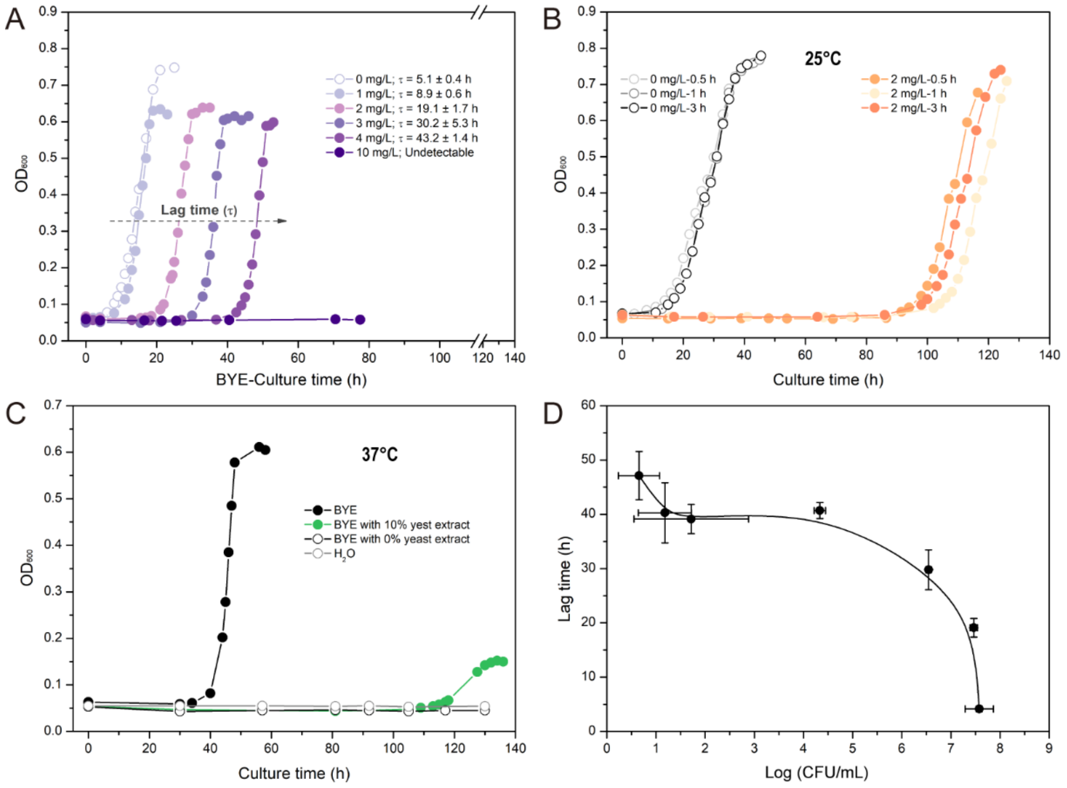

Figure S3. Postchlorination growth behaviors of *L. pneumophila.* (A) Growth behavior in BYE-Culture after exposure to chlorination with CAT dose of 0, 1, 2, 3, 4 and 10 mg/L for 0.5 h. Lag time (τ) increased from 5.1 h (0 mg/L CAT) to > 80 h (10 mg/L CAT). (B) Growth dynamics in BYE medium at 25°C following treatment with 2 mg/L CAT for 0.5, 1, or 3 h. (C) Growth profiles in different BYE media vs. DI-H_2O at 37°C after 6 h exposure to 2 mg/L CAT (closed symbols: chlorinated samples; open circles: DI-H_2O controls). (D) Correlation between τ values (Figure 3A) and the log (CFU/mL) (Figure 3B).

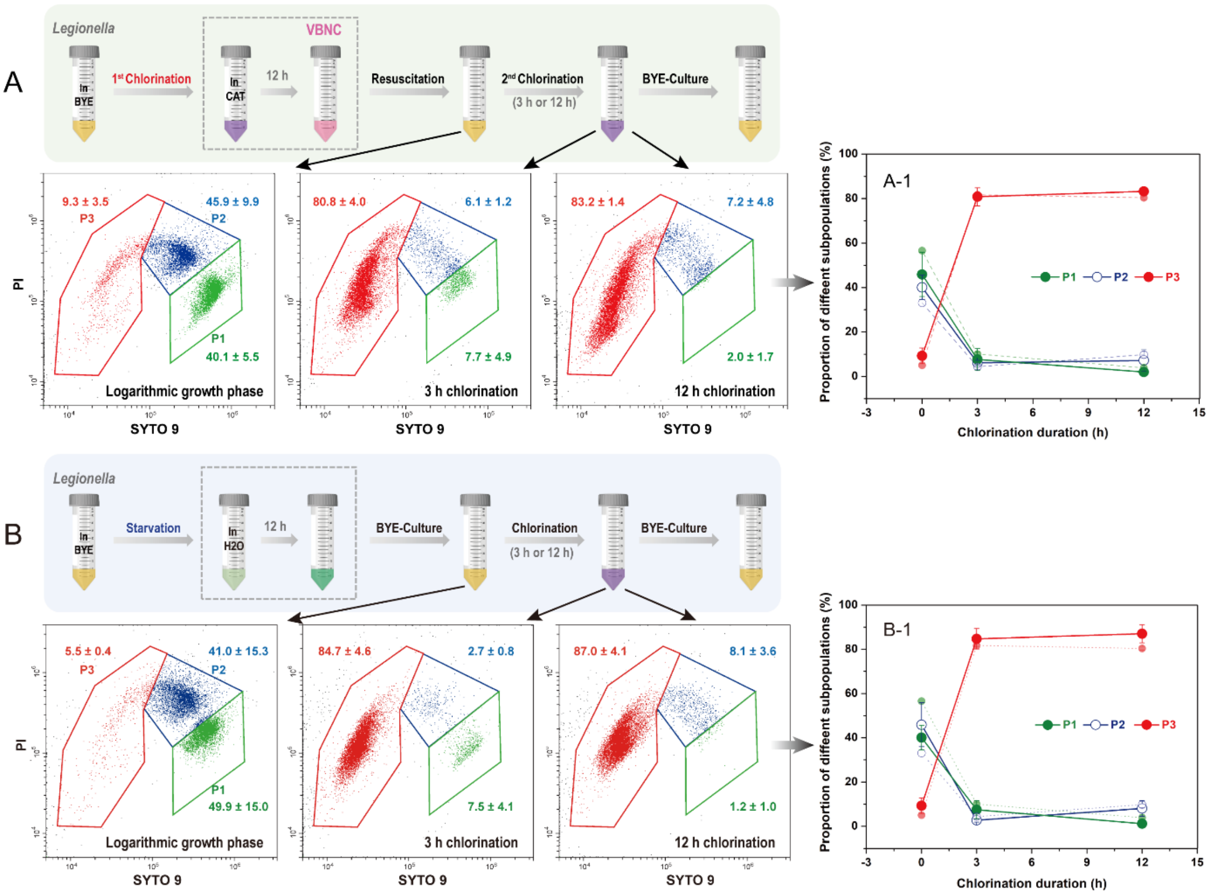

Figure S4. Subpopulation dynamics of *L. pneumophila* under sequential treatments. (A) Single chlorination (2 mg/L CAT, 12 h) followed by resuscitation and repeat chlorination (3 h or 12 h). (B) Parallel workflow substituting initial chlorination with 12 h starvation in DI-H_2O. Representative FCM scatter plots (left to right) show subpopulations at key stages: post-treatment (chlorination/starvation), log-phase resuscitation harvest, and after repeat chlorination (3 h or 12 h). For clarity, Panels A-1 and B-1 compare data from the single chlorination (copied from Figure 2B; small symbols with dashed lines) and repeat chlorination (large symbols with solid lines), where 0 h represents the pretreatment state.

Supplemental Tables

Tables S1–S4 Growth dynamics of *L. pneumophila* (μ: growth rate; τ: lag time).

Table S1. In various BYE media at 25°C or 37°C after inoculating a single colony.

BYE media (Yeast extract%)	25°C		37°C	
	μ (h^{-1})	τ (h)	μ (h^{-1})	τ (h)
10%	0.024 ± 0.005	2.6 ± 2.0	0.038 ± 0.003	2.4 ± 2.3
50%	0.094 ± 0.014	13.0 ± 4.6	0.250 ± 0.028	6.8 ± 1.4
100%	0.104 ± 0.001	17.7 ± 0.1	0.312 ± 0.020	8.3 ± 1.7

Data are shown as mean ± standard deviation (SD).

Table S2. In media with different nutrient levels at 37°C (top) and in BYE-Culture (bottom) after chlorination treatment.

Medium (yeast extract%) [a]	μ (h^{-1})	τ (h)
BYE (10%)	0.071 ± 0.005	112.5 ± 10.1
BYE (100%)	0.307 ± 0.011	39.1 ± 2.7

CAT concentration (mg/L) [b]	μ (h^{-1})	τ (h)
0	0.205 ± 0.005	5.1 ± 0.4
1	0.285 ± 0.017	8.9 ± 0.6
2	0.299 ± 0.006	19.1 ± 1.7
3	0.323 ± 0.010	30.2 ± 5.3
4	0.311 ± 0.008	43.2 ± 1.4

[a] Chlorination (2 mg/L CAT, 6 h); [b] Chlorination duration: 0.5 h; All values are mean ± SD.

Table S3. In BYE medium at 25°C or 37°C after treatment.

Chlorination [a]	25°C		37°C	
	μ (h^{-1})	τ (h)	μ (h^{-1})	τ (h)
0 h	0.120 ± 0.002	10.5 ± 0.4	0.274 ± 0.006	4.2 ± 0.5
0.5 h	0.136 ± 0.002	108.9 ± 14.5	0.299 ± 0.006	19.1 ± 1.7
1 h	0.142 ± 0.002	103.7 ± 21.3	0.313 ± 0.007	29.8 ± 3.7
3 h	0.115 ± 0.013	75.0 ± 19.1	0.297 ± 0.006	40.7 ± 1.5
6 h	0.131 ± 0.009	106.7 ± 12.6	0.307 ± 0.011	39.1 ± 2.7
9 h	n.d.	n.d.	0.309 ± 0.007	40.3 ± 5.5
12 h	n.d.	n.d.	0.305 ± 0.008	47.7 ± 4.0

Starvation [b]	25°C		37°C	
	μ (h^{-1})	τ (h)	μ (h^{-1})	τ (h)
0 h	0.128 ± 0.000	9.3 ± 0.08	0.297 ± 0.003	4.0 ± 0.0
0.5 h	0.133 ± 0.001	11.3 ± 0.02	0.205 ± 0.005	5.1 ± 0.4
1 h	0.145 ± 0.002	13.4 ± 0.5	0.193 ± 0.006	4.7 ± 0.4
3 h	0.131 ± 0.004	12.8 ± 0.0	0.205 ± 0.004	4.7 ± 0.2
6 h	n.d.	n.d.	0.298 ± 0.003	5.8 ± 0.2
9 h	n.d.	n.d.	0.287 ± 0.007	4.8 ± 0.1
12 h	n.d.	n.d.	0.288 ± 0.012	5.1 ± 0.7
16 h	0.115 ± 0.016	17.9 ± 1.2	0.297 ± 0.004	7.7 ± 0.2

[a] 2 mg/L CAT, [b] DI-H_2O; n.d.: not detected. Values are shown as mean ± SD.

Table S4. Untreated *L. pneumophila* or resuscitated *L. pneumophila* in BYE-Culture after chlorination treatment at 2 mg/L CAT.

Duration [a]	μ (h^{-1})	τ (h)		
3 h	0.297 ± 0.006	40.7 ± 1.5		
12 h	0.305 ± 0.008	47.7 ± 4.0		

Duration [b]	Log-phase cells		Stationary-phase cells	
	μ (h^{-1})	τ (h)	μ (h^{-1})	τ (h)
3 h	0.297 ± 0.020	40.9 ± 1.3	0.289 ± 0.030	26.8 ± 3.0
12 h	0.295 ± 0.030	47.6 ± 6.0	0.315 ± 0.070	41.2 ± 0.8

Duration [c]	Log-phase cells		Stationary-phase cells	
	μ (h^{-1})	τ (h)	μ (h^{-1})	τ (h)
3 h	0.327 ± 0.027	46.3 ± 6.4	0.312 ± 0.014	40.6 ± 6.5
12 h	0.327 ± 0.016	43.0 ± 3.3	0.320 ± 0.013	40.6 ± 6.3

[a] Single chlorination; [b] Double chlorination (VBNC-derived cells); [c] Chlorination of starvation-derived cells
All values are shown as mean ± SD from three to seven independent samples.

Supplemental references

1. Fonseca, M. V.; Swanson, M. S. Nutrient salvaging and metabolism by the intracellular pathogen *Legionella pneumophila*. Front Cell Infect Microbiol **2014**, *4*, 12.
2. Devos, L.; Boon, N.; Verstraete, W. *Legionella pneumophila* in the environment: The occurrence of a fastidious bacterium in oligotrophic conditions. Reviews in Environmental Science and Bio-Technology **2005**, *4* (1), 61-74.
3. Manske, C.; Hilbi, H. Metabolism of the vacuolar pathogen *Legionella* and implications for virulence. Frontiers in Cellular and Infection Microbiology **2014**, *4*, 125.
4. Tison, D. L.; Pope, D. H.; Cherry, W. B.; Fliermans, C. B. Growth of *Legionella pneumophila* in association with blue-green algae (cyanobacteria). Applied and Environmental Microbiology **1980**, *39* (2), 456-459.
5. Surman, S. B.; Morton, L. H. G.; Keevil, C. W. The dependence of *Legionella pneumophila* on other aquatic bacteria for survival on R2A medium. International Biodeterioration & Biodegradation **1994**, *33* (3), 223-236.
6. Dutil, S.; Tessier, S.; Veillette, M.; Laflamme, C.; Meriaux, A.; Leduc, A.; Barbeau, J.; Duchaine, C. Detection of *Legionella spp.* by fluorescent in situ hybridization in dental unit waterlines. Journal of Applied Microbiology **2006**, *100* (5), 955-963.
7. States, S. J.; Wadowsky, R. M.; Kuchta, J. M.; Wolford, R. S.; Conley, L. F.; Yee, R. B. *Legionella* in drinking water. In Drinking Water Microbiology: Progress and Recent Developments, McFeters, G. A., Ed. (Springer New York), 1990; pp 340-367.
8. Gao, H.; Liu, C. C. Biochemical and morphological alteration of *Listeria monocytogenes* under environmental stress caused by chloramine-T and sodium hypochlorite. Food Control **2014**, *46*, 455-461.
9. Votyakova, T. V.; Kaprelyants, A. S.; Kell, D. B. Influence of viable cells on the resuscitation of dormant cells in *Micrococcus luteus* cultures held in an extended stationary phase: the population effect. Applied and Environmental Microbiology **1994**, *60* (9), 3284-91.
10. Chang, C. W.; Hwang, Y. H.; Cheng, W. Y.; Chang, C. P. Effects of chlorination and heat disinfection on long-term starved *Legionella pneumophila* in warm water. Journal of Applied Microbiology **2007**, *102* (6), 1636-1644.
11. Cervero-Aragó, S.; Schrammel, B.; Dietersdorfer, E.; Sommer, R.; Lueck, C.; Walochnik, J.; Kirschner, A. Viability and infectivity of viable but nonculturable *Legionella pneumophila* strains induced at high temperatures. Water Research **2019**, *158*, 268-279.
12. Li, L.; Mendis, N.; Trigui, H.; Faucher, S. P. Transcriptomic changes of *Legionella pneumophila* in water. BMC Genomics **2015**, *16*, 637.
13. Al-Bana, B. H.; Haddad, M. T.; Garduño, R. A. Stationary phase and mature infectious forms of *Legionella pneumophila* produce distinct viable but non-culturable cells. Environmental Microbiology **2014**, *16* (2), 382-395.
14. Hammes, F.; Berney, M.; Wang, Y.; Vital, M.; Köster, O.; Egli, T. Flow-cytometric total bacterial cell counts as a descriptive microbiological parameter for drinking water treatment processes. Water Research **2008**, *42* (1), 269-277.
